# *In Vitro* Antifungal Activity of Manogepix and Other Antifungal Agents against South African *Candida auris* Isolates from Bloodstream Infections

**DOI:** 10.1128/spectrum.01717-21

**Published:** 2022-02-23

**Authors:** Tsidiso G. Maphanga, Ruth S. Mpembe, Serisha D. Naicker, Nelesh P. Govender, for GERMS-SA

**Affiliations:** a National Institute for Communicable Diseases (Centre for Healthcare-Associated Infections, Antimicrobial Resistance and Mycoses), National Health Laboratory Service, Johannesburg, South Africa; b School of Pathology, Faculty of Health Sciences, University of the Witwatersrand, Johannesburg, South Africa; c Division of Medical Microbiology, Faculty of Health Sciences, University of Cape Town, Cape Town, South Africa; d Medical Research Council Centre for Medical Mycology, College of Medicine and Health, University of Exeter, Exeter, United Kingdom; University of Lagos

**Keywords:** *Candida auris*, manogepix, fosmanogepix, antifungal susceptibility, candidemia, multidrug-resistant, panfungal-resistant

## Abstract

We determined the susceptibility of South African Candida auris bloodstream surveillance isolates to manogepix, a novel antifungal, and several registered antifungal agents. C. auris isolates were submitted to a reference laboratory between 2016 and 2017. Species identification was confirmed by phenotypic methods. We determined MICs for amphotericin B, anidulafungin, caspofungin, micafungin, itraconazole, posaconazole, voriconazole, fluconazole, and flucytosine using Sensititre YeastOne and manogepix using a modified Clinical and Laboratory Standards Institute broth microdilution method. Clade distribution was determined for a subset of isolates using whole-genome sequencing. Of 394 tested isolates, 357 were resistant to at least 1 antifungal class. The manogepix MIC range was 0.002 to 0.06 μg/mL for 335 isolates with fluconazole monoresistance. Nineteen isolates were resistant to both fluconazole and amphotericin B yet still had low manogepix MICs (range, 0.004 to 0.03 μg/mL). Two isolates from the same patient were panresistant but had manogepix MICs of 0.004 μg/mL and 0.008 μg/mL. Comparing MIC_50_ values, manogepix was >3-fold more potent than azoles, 4-fold more potent than echinocandins, and 9-fold more potent than amphotericin B. Of 84 sequenced isolates, the manogepix MIC range for 70 clade III isolates was 0.002 to 0.031 μg/mL, for 13 clade I isolates was 0.008 to 0.031 μg/mL, and for one clade IV isolate, 0.016 μg/mL. Manogepix exhibited potent activity against all isolates, including those resistant to more than one antifungal agent and in three different clades. These data support manogepix as a promising candidate for treatment of C. auris infections.

**IMPORTANCE** Since C. auris was first detected in South Africa in 2012, health care-associated transmission events and large outbreaks have led to this pathogen accounting for more than 1 in 10 cases of candidemia. A large proportion of South African C. auris isolates are highly resistant to fluconazole but variably resistant to amphotericin B and echinocandins. There is also an emergence of pandrug-resistant C. auris isolates, limiting treatment options. Therefore, the development of new antifungal agents such as fosmanogepix or the use of new combinations of antifungal agents is imperative to the continued effective treatment of C. auris infections. Manogepix, the active moiety of fosmanogepix, has shown excellent activity against C. auris isolates. With the emergence of C. auris isolates that are pandrug-resistant in South Africa, our *in vitro* susceptibility data support manogepix as a promising new drug candidate for treatment of C. auris and difficult-to-treat C. auris infections.

## INTRODUCTION

Candida auris is an important cause of invasive infections in both acute and long-term health care settings and as of February 2021 had been reported in more than 47 countries (1). This fungal pathogen colonizes both skin and mucosal surfaces, forms biofilms, is resistant to some standard disinfectant solutions, and is transmitted by contact (2). C. auris causes outbreaks of infection, and particularly among critically ill and immunosuppressed patients, invasive infection is associated with high mortality (3–5). The coronavirus disease (COVID-19) pandemic has led to a global surge in hospitalizations with an increasing number of critically ill people at risk for health care-associated infections caused by C. auris (6). In laboratories using older methods of identification, C. auris can still be misidentified and is resistant to multiple antifungal classes based on tentative breakpoints (7, 8). Through whole-genome sequencing (WGS), C. auris was grouped into four genotypic clades named for their geographic origin: clade I (South Asia), clade II (East Asia), clade III (Africa), and clade IV (South America) (9–11). A clade V isolate, which differs from the other four by >200,000 single nucleotide polymorphisms (SNP), was cultured from a 14-year-old girl with otomycosis in Iran (3). The four main clades differ in their antifungal resistance profiles, with clade II being less resistant than clades I, III, and IV (10, 12).

In South Africa, C. auris is the third most common *Candida* species causing candidemia (13). Echinocandins are recommended for treatment of bloodstream C. auris infection (14, 15). A large proportion of South African C. auris isolates, dominated by clade III, are resistant to fluconazole but variably resistant to amphotericin B and echinocandins (16). For comparison, 90% of 350 Indian C. auris isolates collected between 2009 and 2017 and dominated by clade I were resistant to fluconazole and lower proportions were resistant to echinocandins (2%) and amphotericin B (8%) (17). Furthermore, the emergence of pandrug-resistant C. auris isolates limits treatment options (18). Therefore, the development of new antifungal agents or the use of new combinations of antifungal agents is imperative to the continued effective treatment of C. auris infections. Several antifungal agents with novel mechanisms of action and potent *in vitro* activity against C. auris are in the pipeline (19). Among these is fosmanogepix, a first-in-class small-molecule antifungal agent which is currently in phase 2 clinical trials for the treatment of invasive fungal infections (20). The active moiety, manogepix, is an inhibitor of glycosylphosphatidylinositol (GPI) biosynthesis. Manogepix targets the highly conserved Gwt1 enzyme, thereby blocking GPI posttranslational modification, which is necessary for the anchoring of GPI-anchored surface proteins to the fungal cell wall (20). Several studies have reported excellent *in vitro* activity of manogepix against C. auris and other fungi causing invasive infections, including isolates which were resistant to more than one antifungal agent (20–26). In order to determine the activity of manogepix and other registered antifungal agents against South African isolates, we performed *in vitro* antifungal susceptibility of C. auris bloodstream isolates collected through a national laboratory surveillance program in 2016 to 2017.

## RESULTS

### Selection of isolates and cases.

Between 2016 and 2017, 400 C. auris isolates from 344 cases were submitted to NICD as part of candidemia surveillance. Of the 400 isolates, 257 isolates were correctly identified as C. auris by the submitting laboratories. Of the 137 with an incorrect identification at the submitting laboratory, 53 were identified to species level (Candida haemulonii [*n* = 34], Saccharomyces cerevisiae [*n* = 7], Candida parapsilosis [*n* = 5], Candida albicans [*n* = 4], *Nakaseomyces glabrata* [*n* = 2], and Candida lusitaniae [*n* = 1]). The remaining 84 isolates were not identified to species level. Of the 400 isolates confirmed as C. auris at NICD, 394 isolates cultured from 340 cases had manogepix MICs determined; the remaining six isolates were contaminated during storage before manogepix MICs could be determined. Of the 340 cases, 45 cases had more than one isolate tested (16).

### Distribution of MICs.

The broth microdilution (BMD) and Etest MIC distribution, MIC_50_, and MIC_90_ of 10 antifungal agents for the 394 C. auris isolates are presented in Table 1. The BMD MIC_50_ and MIC_90_ values for all tested isolates of manogepix were 0.008 μg/mL and 0.016 μg/mL, which were lower than those of all other antifungal agents tested. Of the 394 C. auris isolates, 357 fluconazole-resistant isolates had a manogepix MIC range of 0.002 μg/mL to 0.063 μg/mL, while 37 fluconazole-susceptible isolates had a manogepix MIC range of 0.002 μg/mL to 0.031 μg/mL. Comparing manogepix BMD MIC_90_ values to those of the other antifungal agents, manogepix was 3- to 6-fold more potent than itraconazole, posaconazole, voriconazole, and fluconazole, 4-fold more potent than micafungin and anidulafungin, and 9-fold more potent than amphotericin B. According to the ECOFFFinder results, the wild-type upper limit (WT-UL) MIC for manogepix, using the 99.0% cutoff value, was 0.06 μg/mL. At this cutoff value, there were no non-WT isolates for manogepix.

**TABLE 1:** MIC distribution of 394 South African C. auris bloodstream isolates, 2016 to 2017^*a*^

Antifungal agent	Test method	No. of isolates with MIC (μg/mL) of:																			GM MIC	Mode	MIC_50_	MIC_90_	MIC range	% Resistant
		0.002	0.004	0.008	0.015	0.03	0.06	0.125	0.25	0.5	1	2	3	4	8	16	32	64	128	256						
Manogepix	BMD	10	76	208	85	14	1														0.008	0.008	0.008	0.016	0.002–0.06	
Itraconazole	BMD					3	53	202	119	12	3	2									1.32	0.125	0.12	0.25	0.03–2	
Voriconazole	BMD					3	8	18	50	119	140	54		1	1						1.23	1	0.5	2	0.03–8	
Posaconazole	BMD			2	24	90	154	102	18	3	1										1.00	0.06	0.06	0.12	0.008–1	
Fluconazole	BMD													3	5	30	**56**	**81**	**109**	**110**	88.85	256	128	256	4–256	90
Caspofungin	BMD			3	2	21	195	128	26	12	3				1	2					3.56	0.06	0.06	0.25	0.008–16	
Micafungin	BMD			2		12	195	156	14	8	4	1		**1**	**1**						1.18	0.06	0.06	0.12	0.008–8	0.5
Anidulafungin	BMD				2	4	92	230	53	7	5	1									0.82	0.125	0.12	0.25	0.015–2	0
Flucytosine	BMD			1	1		78	254	55	3	1	1									1.41	0.125	0.12	0.25	0.008–2	
Amphotericin B	BMD								1	21	265	**104**			**3**						1.23	1	1	2	0.25–8	27
Micafungin	Etest															**2**										0.5
Amphotericin B	Etest	7	3	2	2	4	9	28	120	143	54	**15**	**2**	**4**	**1**						1.63	0.5	0.38	1	0.002–8	6

a BMD, broth microdilution; GM, geometric mean; MIC, minimum inhibitory concentration. Resistant isolates are highlighted in bold. Tentative breakpoints: fluconazole ≥ 32 μg/mL, micafungin and anidulafungin ≥ 4 μg/mL, amphotericin B ≥ 2 μg/mL.

### Manogepix activity against resistant C. auris isolates.

**FIG 1:**
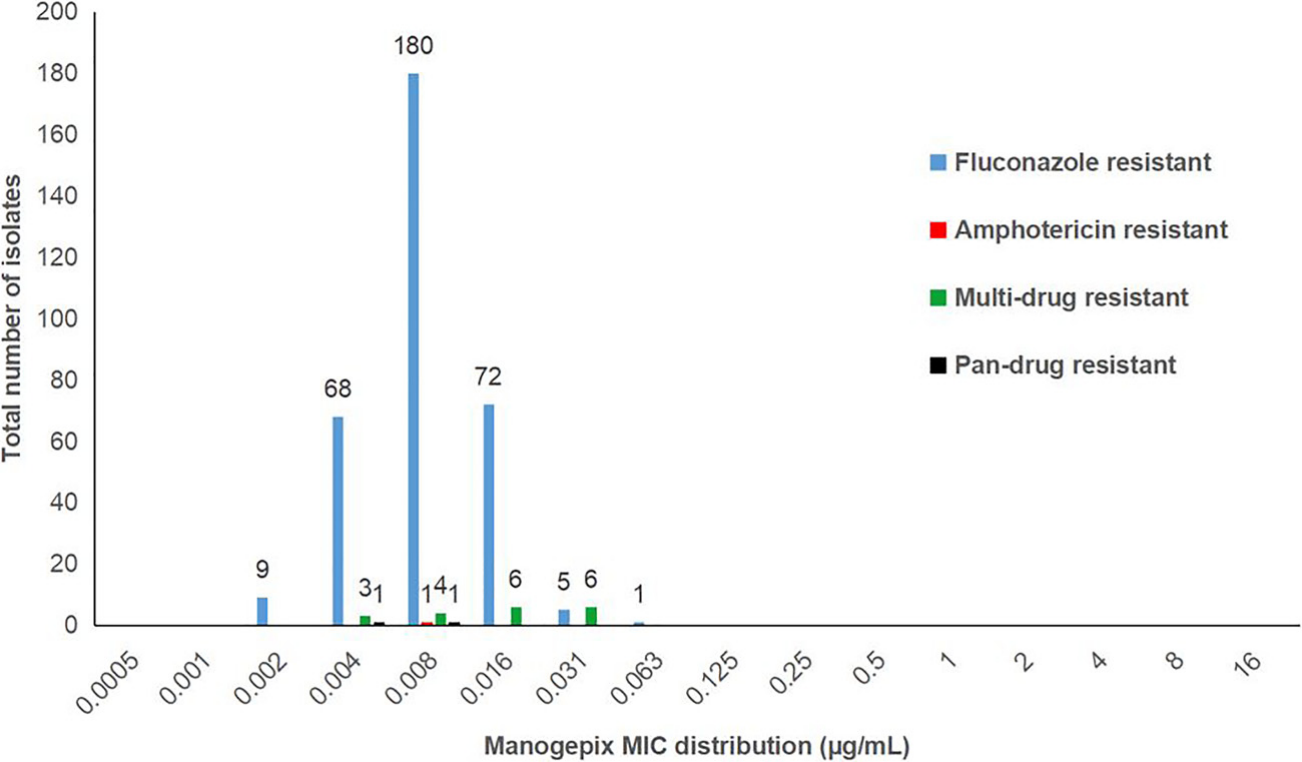
Manogepix MICs distribution for mono-, multi-, and pandrug-resistant C. auris isolates (*n* = 357), South Africa, 2016 to 2017. Fluconazole MIC ≥ 32 μg/mL, micafungin MIC ≥ 4 μg/mL, amphotericin B MIC ≥ 2 μg/mL.

Of 394 C. auris isolates, 357 (91%) were resistant to at least 1 antifungal class (Fig. 1). A total of 335 C. auris isolates were resistant to fluconazole alone with BMD MIC_50_ of 128 μg/mL and MIC_90_ of 256 μg/mL. The manogepix BMD MIC_50_ and MIC_90_ values for these isolates were 0.008 μg/mL and 0.016 μg/mL, with an MIC range of 0.002 μg/mL to 0.063 μg/mL. A single amphotericin B mono-resistant isolate had a manogepix MIC of 0.008 μg/mL. Nineteen isolates, which were resistant to both fluconazole and amphotericin B, had low manogepix MICs (range, 0.004 μg/mL to 0.031 μg/mL) (Fig. 1). Two isolates from the same patient were resistant to all three antifungal classes. These two isolates had micafungin Etest MIC of 16 μg/mL, fluconazole BMD MICs of 32 μg/mL and 64 μg/mL, and amphotericin B Etest MICs of 4 μg/mL and 2 μg/mL. The manogepix MICs for these two isolates were 0.004 μg/mL and 0.008 μg/mL, respectively.

### Manogepix activity across C. auris clades.

**FIG 2:**
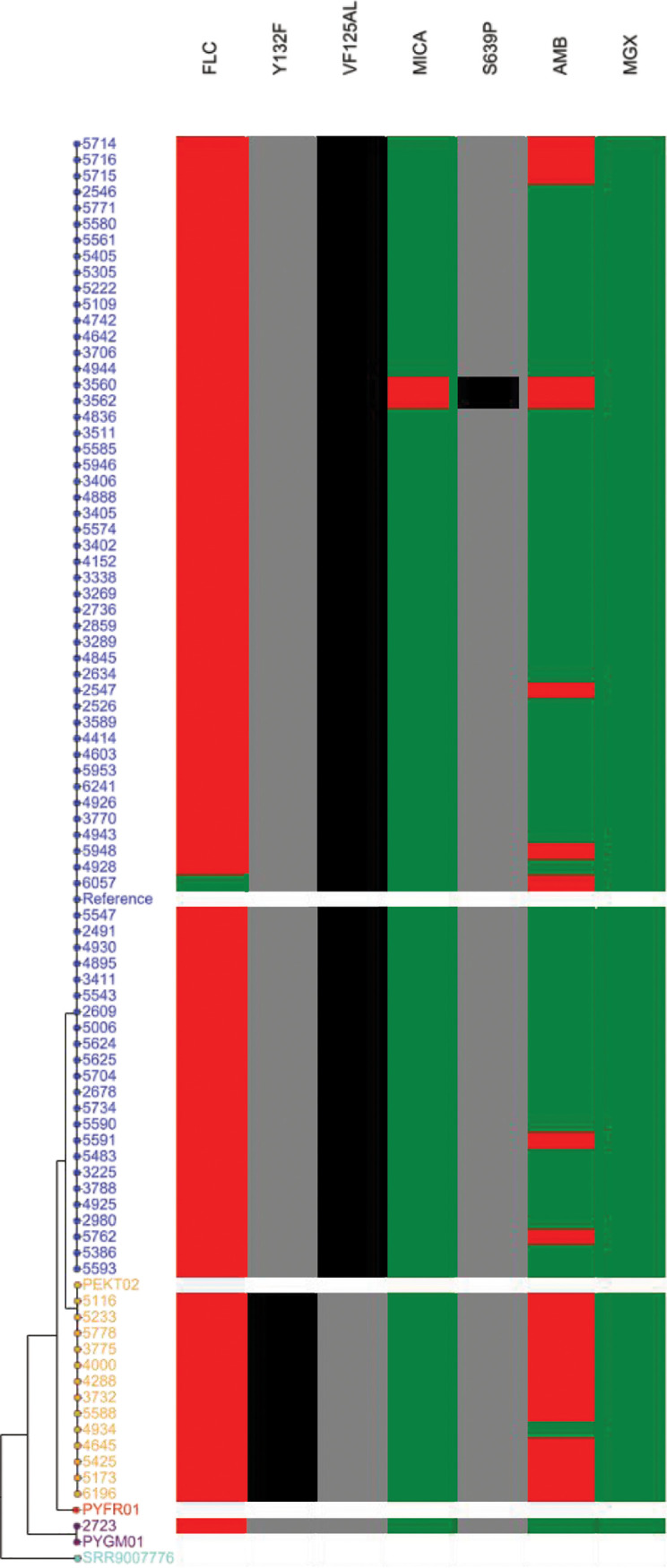
Whole-genome sequencing single nucleotide polymorphism analysis of 84 South African bloodstream C. auris isolates collected between 2016 to 2017 during national laboratory-based candidemia surveillance. The phylogenetic tree shows the relationship of isolates by clade and their susceptibility to fluconazole (FLC), micafungin (MICA), amphotericin B (AMB), and manogepix (MGX) with corresponding point mutations in the *ERG11* gene (Y132F, VF125AL) associated with azole resistance, and the *FKS1* gene hot spot 1 (S639P) associated with echinocandin resistance. Blue, clade III isolates; orange, clade I isolates; purple, clade IV isolate; turguoise, clade V reference isolate; red, clade II reference isolate; red, resistant isolates (FLC: ≥32 μg/mL, MICA: ≥4 μg/mL, AMB: ≥2 μg/mL); green, susceptible isolates (FLC: ≤32 μg/mL, MICA: ≤4 μg/mL, AMB: ≤2 μg/mL, MGX: ≤0.016 μg/mL); gray, mutation absent; black, mutation present.

**FIG 3:**
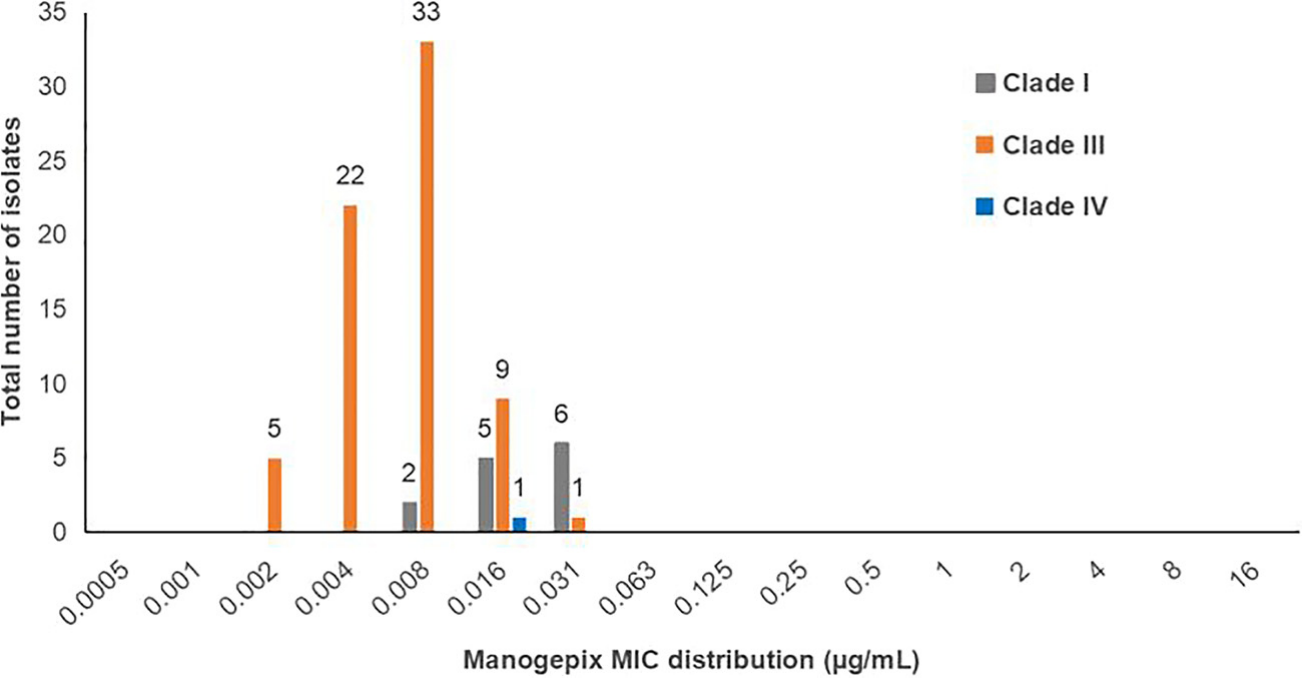
Manogepix MICs distribution across different clades of C. auris isolates (*n* = 84), 2016 to 2017. Seventy isolates belonged to clade III, 13 to clade I, and 1 to clade IV.

Of the 84 sequenced C. auris isolates, 70 belonged to clade III, 13 to clade I, and 1 to clade IV (Fig. 2). These isolates were all resistant to at least one antifungal agent. All 84 sequenced C. auris isolates had low manogepix MICs irrespective of their clade (Fig. 3). The 70 resistant clade III isolates had a manogepix BMD MIC_50_ of 0.008 μg/mL and MIC_90_ of 0.016 μg/mL with an MIC range of 0.002 μg/mL to 0.031 μg/mL. Among the 13 resistant clade I isolates, the manogepix BMD MIC_50_ was 0.016 μg/mL and the MIC_90_ was 0.03 μg/mL with an MIC range of 0.008 μg/mL to 0.031 μg/mL. The fluconazole-resistant clade IV isolate had a manogepix BMD MIC of 0.016 μg/mL (Fig. 3). The clade III and I isolates had VF125AL and Y132F amino acid substitutions in Erg11p, respectively (Fig. 2). The two echinocandin-resistant clade III isolates had the S639P substitution in Fks1p.

## DISCUSSION

We compared the antifungal susceptibility of a novel antifungal agent, manogepix, and several registered antifungal agents against 394 South African C. auris isolates from episodes of bloodstream infection. Based on comparisons of the MIC_90_ values, manogepix was more potent than the azoles, echinocandins, and amphotericin B. This novel antifungal agent was also active against multidrug-resistant and pandrug-resistant C. auris isolates. Manogepix was active against 84 sequenced isolates from three different clades, all of which were resistant to at least 1 other antifungal agent, and retained activity against those with resistance gene mutations.

C. auris is responsible for approximately 1 in 10 cases of candidemia in South Africa and has been associated mostly with large outbreaks in both public- and private-sector hospitals (14). Although only 5% and 1% of the C. auris bloodstream isolates were multidrug- and pandrug-resistant, respectively, any extensive drug resistance in a clinical setting is of major concern since this limits treatment options and there is potential for clonal expansion. Pandrug-resistant C. auris strains have also been reported in Kenya, the United Arab Emirates, and the United States (12). Novel antifungal agents such as ibrexafungerp (triterpenoid class), VT-1598 (tetrazole class), and fosmanogepix, the prodrug of manogepix, may prove useful in management of difficult-to-treat C. auris infections (19). Fosmanogepix is in phase II clinical trials for the treatment of invasive candidiasis, aspergillosis, and rare mold infections (https://clinicaltrials.gov/, identifiers NCT03604705, NCT04240886, NCT04148287) (27). Fosmanogepix differs from other antifungal classes in that it is a novel N-phosphonooxymethyl prodrug that can be quickly and completely metabolized by host systemic phosphatases to the active moiety, manogepix (22, 28). This active moiety then targets the fungal inositol acyltransferase enzyme GWT1, thereby preventing GPI-anchored protein maturation and compromising fungal growth (22, 28).

All the C. auris isolates in this study, most of which belonged to clade III, had low manogepix MICs regardless of whether these isolates were resistant to fluconazole, amphotericin B, or echinocandins. This is consistent with what has previously been reported from *in vitro* studies of manogepix tested against C. auris isolates from different geographic areas (21). Sixteen C. auris isolates from Germany, Japan, South Korea, and India had a manogepix MIC_90_ of 0.031 μg/mL versus a fluconazole MIC_90_ of >64 μg/mL, amphotericin B MIC_90_ of 3 μg/mL, and micafungin MIC_90_ of 2 μg/mL (21). Another study from an outbreak in the United States also reported excellent activity of manogepix against 200 C. auris isolates (24). In the latter Zhu et al. study, the manogepix MIC_90_ of 0.031 μg/mL was lower than the fluconazole MIC_90_ of 256 μg/mL, amphotericin B MIC_90_ of 2 μg/mL, and micafungin MIC_90_ of 0.25 μg/mL (24). The manogepix MIC_90_ in our study (0.016 μg/mL) was one dilution lower. We also found that no C. auris isolates were classified as non-WT for manogepix at the upper limit of 0.06 μg/mL, which is similar to the findings of Zhu et al. (24). While Arendrup et al. found that isolates with high fluconazole MICs also had high manogepix MICs and those with low fluconazole MICs had low manogepix MICs, we found no difference in manogepix activity among fluconazole-resistant or -susceptible isolates (22). It is possible that the activity of manogepix is not affected by all the mechanisms of fluconazole resistance (24). Of 83 fluconazole-resistant C. auris isolates and a single fluconazole-susceptible isolate which had their genomes sequenced, as well as 35 fluconazole-susceptible isolates for which WGS was not analyzed in the current study, all had mutations in the *ERG11* genes. All these isolates also had low manogepix MICs with no differences in manogepix MIC between fluconazole-resistant and -susceptible isolates (16).

We observed no differences in manogepix MICs between multidrug- and pandrug-resistant isolates. The action of manogepix against multidrug-resistant and panresistant C. auris isolates is probably not affected by the genetic mechanisms of resistance to azoles, polyenes, and echinocandins in these isolates. Although the azoles and polyenes, like manogepix, affect the integrity of the fungal cell membrane, these antifungals target different enzymes (29). This is supported by the two pandrug-resistant isolates with manogepix MICs of 0.004 μg/mL and 0.008 μg/mL in our study. Berkow and Lockhart and Zhu et al. also tested two and six pandrug-resistant isolates and reported manogepix MICs of 0.004 μg/mL to 0.008 μg/mL and 0.008 μg/mL to 0.016 μg/mL, respectively (24, 29). Our C. auris isolates clustered into three different clades. With phylo-geographic mixing, C. auris outbreaks from Canada, Kenya, and the United States have also been reported to comprise multiple clades (12). Chow et al. found 45% of clade I isolates to be multidrug-resistant versus clade III (8%) and clade IV (10%) using the Etest method (12). We also observed a high percentage (92%) of multidrug resistance among clade I isolates versus clade III isolates (9%). Comparing manogepix activity among the three different clades, the manogepix MICs were low irrespective of the clade. Berkow and Lockhart tested 100 C. auris isolates from different geographic areas/clades and also reported a manogepix MIC range of <0.0005 μg/mL to 0.015 μg/mL with no differences in activity between isolates from the different clades (28). Only six clade I isolates (most multidrug resistant) and one clade III isolate had an MIC of 0.031 μg/mL, which is one dilution higher than that reported by Berkow and Lockhart. Five isolates with no assigned clade had a manogepix MIC of 0.031 μg/mL. Of the 122 Indian isolates (clade I) in the Arendrup et al. study, a majority (65%) of the isolates had a manogepix MIC of 0.008 to 0.031 μg/mL as determined by CLSI method (22). Eleven isolates representing the South Asian (*n* = 5) and South American (*n* = 6) clades were also inhibited by manogepix MIC of ≤0.06 μg/mL (25).

A strength of our study is that we tested a large number of C. auris isolates from national surveillance and compared the manogepix MICs to those of other registered antifungal agents in South Africa. We used the BMD method for manogepix testing, which allowed for accurate comparisons with previously published studies (20, 21, 23–25, 29). We found that manogepix had excellent activity against resistant C. auris isolates and could thus be useful for treatment of difficult-to-treat infections. Although we have confirmed the *in vitro* activity of manogepix against C. auris isolates, clinical trials are needed to understand the pharmacokinetics and pharmacodynamics of this novel agent as well as safety and efficacy in patients with C. auris infections. A limitation of the study is that we did not assign all 394 C. auris isolates to a clade, although we did use random sampling based on phenotypic resistance patterns to limit selection bias.

**Conclusions.** Manogepix MICs were lower than those of other antifungal agents in a large collection of South African C. auris bloodstream isolates. This antifungal agent also had potent activity against multidrug-resistant and panresistant C. auris isolates irrespective of the clade or presence of resistance gene mutations. Manogepix is a promising new drug candidate for treatment of C. auris infections.

## MATERIALS AND METHODS

### National surveillance and case definition.

Clinical Candida auris isolates were collected during national laboratory-based surveillance conducted from 1 January 2016 through 31 December 2017 at laboratories affiliated with the National Health Laboratory Service (NHLS) or private pathology practices in South Africa. We defined a case as a person of any age with a positive C. auris blood culture indicating a bloodstream infection. Laboratories were requested to submit all *Candida* species isolated from blood cultures to the National Institute for Communicable Diseases (NICD) in Johannesburg and to provide the corresponding patient demographic details and *Candida* species identification obtained by the submitting laboratory. The isolate data in this study were published previously (16, 30).

### Confirmation of C. auris.

*Candida* isolates were stored at −70°C after species identification was performed at the NICD by previously described phenotypic methods (15). For this study, we retrieved the stored C. auris isolates and subcultured them on chromogenic agar (CHROMagar Candida, Mast Diagnostics, Amiens, France) for a purity check. Species identification of single colonies was then reconfirmed using matrix-assisted laser desorption/ionization time-of-flight mass spectrometry (MALDI-TOF MS) (Bruker Corporation, Billerica, MA, United States). Isolates that were contaminated in storage were excluded.

### Antifungal susceptibility testing.

**(i) Manogepix.** We tested the activity of manogepix (MGX, APX001A) against all available C. auris strains. The manogepix powder was supplied by Amplyx Pharmaceuticals Inc., San Diego, California. MICs for manogepix were determined using broth microdilution (BMD) panels prepared at NICD following Clinical and Laboratory Standards Institute (CLSI) M27-Ed4 recommendations with one modification (31). Briefly, panels were made using RPMI 1640 broth supplemented with morpholinepropane-sulfonic acid (MOPS) buffer and 0.2% glucose. A manogepix stock solution of 10 mg/mL was prepared in 100% dimethyl sulfoxide (DMSO), as recommended by the manufacturer. The 15-μL aliquots of the stock solution were kept at −70°C. We then prepared intermediate dilutions of the manogepix stock using DMSO to obtain final concentrations of 0.0005 μg/mL to 16 μg/mL. One microliter of the manogepix solution, instead of 100 μL as per CLSI recommendations, was added to microtiter plates, and RPMI broth containing a final concentration of 2.5 × 10^3^ cells/mL was then added. A total of 1 μL of DMSO was also added to “no drug” control wells. The manufacturer-recommended manogepix MIC range is 0.001 μg/mL to 2 μg/mL. All plates were incubated at 35°C and MICs were visually evaluated for growth following 24 h of incubation. The MIC was defined as the lowest manogepix concentration that caused ≥50% growth inhibition compared to the positive growth control as per manufacturer recommendations. C. parapsilosis ATCC 22019 and Candida albicans ATCC 90028 were run on all days of testing, and MICs were found to be within the required quality control ranges (0.008 to 0.03 mg/L for ATCC 22019 and 0.004 to 0.015 mg/L for ATCC 90028).

**(ii) Other antifungal agents.** The MICs for nine other antifungal agents (i.e., amphotericin B, fluconazole, voriconazole, itraconazole, posaconazole, caspofungin, anidulafungin, micafungin, and flucytosine) were determined using a commercial broth microdilution method (Sensititre YeastOne, Thermo Fisher Scientific, Cleveland, OH, USA) as per manufacturer’s instructions. The echinocandin, azole, and flucytosine MICs were read at 50% growth inhibition compared to the positive control, while the MICs for amphotericin B were read at 100% inhibition as per CLSI recommendations (31). We used CDC tentative breakpoints to define resistance in C. auris: amphotericin B MIC of ≥2 μg/mL, fluconazole MIC of ≥32 μg/mL, and anidulafungin/micafungin MIC of ≥4 μg/mL. We did not interpret caspofungin MICs; instead, micafungin or anidulafungin resistance was used as a surrogate marker of resistance to the entire class (32). Multidrug resistance was defined as resistance to two antifungal classes, while pandrug resistance was defined as resistance to three antifungal classes. There are no breakpoints to interpret itraconazole, posaconazole, voriconazole, and flucytosine MICs. Quality control strains of C. parapsilosis ATCC 22019 and Candida krusei ATCC 6258 were included in all runs as described above. Amphotericin B MICs were also determined by gradient diffusion strips (Etest, bioMérieux, Marcy l’Etoile, France) according to the manufacturer’s instructions. In addition, gradient diffusion MICs were determined for all isolates and found to be echinocandin-resistant by Sensititre (16).

The MIC_50_, MIC_90_, and ranges were calculated for all 10 antifungal agents. There are currently no published breakpoints or epidemiological cutoff (ECV) values for manogepix against C. auris isolates. We used the ECOFFinder program XL 2010 v2.1 (obtained from https://clsi.org/meetings/microbiology/ecoffinder/) to calculate the wild-type upper limit (WT-UL) (33). This was defined as the upper MIC where the wild-type distribution ends and corresponded to approximately 99.0% of the MIC distribution (26). We used the WT-UL to define the wild-type (MIC ≤ WT-UL) and non-wild-type (MIC ≥ WT-UL) populations for manogepix (26, 33).

### Phylogenetic analysis of resistant C. auris isolates.

We used the sequenced genomes of 84 C. auris isolates to perform a phylogenetic and resistance mutation analysis, as described previously (16, 30). Of these 84 isolates, 62 were resistant to fluconazole alone, 19 were multidrug resistant (i.e., resistant to fluconazole and amphotericin B), 2 were pandrug resistant (i.e., resistant to fluconazole, amphotericin B, and micafungin), and 1 was resistant to amphotericin B alone. DNA extraction and paired-end libraries were prepared as described previously (16). We used FastQC and PRINSEQ to assess the quality of the read data and to perform read filtering for sequences of low quality. We aligned the paired-end reads data to a South African C. auris strain (B11221), which had been previously sequenced on the PacBio platform by Lockhart et al. in 2017, using Burrows-Wheeler Aligner (BWA) (9). We included reference genome strains representing clade I (PEKT02), clade II (PYFR01), clade IV (PYGM01), and clade V (SRR9007776), which we obtained from NCBI BLAST. Single nucleotide polymorphism variants were called using the Northern Arizona Single Nucleotide Polymorphism (NASP) pipeline, which is publicly available, as described previously (30). Phylogenetic analysis was performed using MEGA software. We previously assessed the presence of mutations using a target gene approach on CLC Genomics Workbench version 10 (Qiagen, The Netherlands) (16). For the 84 resistant isolates, we looked for mutations within the *ERG11* gene (associated with azole resistance) and *FKS1* hot spot 1 region (associated with echinocandin resistance). We aligned these isolates to a reference wild-type genome of clade I C. auris B8441 (GenBank accession PEKT00000000.2) to detect mutant genes within the *ERG11* and *FKS1HS1* genes. Sequences were examined visually and changes within the DNA sequences were noted and compared to those previously reported by Lockhart et al. and Chow et al. (9, 12).

### Ethics approval.

Ethical approval for GERMS-SA laboratory-based surveillance was obtained through research ethics committees of several South African universities (University of Pretoria, University of the Free State, University of the Witwatersrand, University of KwaZulu-Natal, University of Cape Town, Stellenbosch University, and Sefako Makgatho University).

### Data availability.

All WGS data are in the NCBI SRA under BioProject PRJNA737309 with the following sequences accession numbers: SAMN19689606, SAMN19689548, SAMN19689546, SAMN19689594, SAMN19689592, SAMN19689604, SAMN19689585, SAMN19689619, SAMN19689600, SAMN19689601, SAMN19689621, SAMN19689547, SAMN19689583, SAMN19689584, SAMN19689603, SAMN19689599, SAMN19689598, SAMN19689590, SAMN19689544, SAMN19689625, SAMN19689616, SAMN19689572, SAMN19689611, SAMN19689620, SAMN19689549, SAMN19689560, SAMN19689622, SAMN19689571, SAMN19689569, SAMN19689580, SAMN19689561, SAMN19689518, SAMN19689581, SAMN19689519, SAMN19689566, SAMN19689586, SAMN19689605, SAMN19689624, SAMN19689609, SAMN19689607, SAMN19689613, SAMN19689563, SAMN19689562, SAMN19689582, SAMN19689565, SAMN19689564, SAMN19689610, SAMN19689627, SAMN19689574, SAMN19689575, SAMN19689631, SAMN19689595, SAMN19689557, SAMN19689618, SAMN19689576, SAMN19689589, SAMN19689545, SAMN19689556, SAMN19689550, SAMN19689570, SAMN19689612, SAMN19689597, SAMN19689552, SAMN19689623, SAMN19689573, SAMN19689543, SAMN19689617, SAMN19689555, SAMN19689591, SAMN19689559, SAMN19689568, SAMN19689520, SAMN19689608, SAMN19689626, SAMN19689628, SAMN19689629, SAMN19689630, SAMN19689587, SAMN19689551, SAMN19689553, SAMN19689577, SAMN19689615, SAMN19689578, SAMN19689558.

## References

[1] Centers for Disease Control and Prevention (CDC). Tracking *Candida auris*, countries from which *Candida auris* cases have been reported, as of February 15, 2021. https://www.cdc.gov/fungal/candida-auris/tracking-c-auris.html#historical.

[2] Ku TSN, Walraven CJ, Lee SA. 2018. *Candida auris*: disinfectants and implications for infection control. Front Microbiol 9:726. doi:10.3389/fmicb.2018.00726. 29706945 PMC5906573

[3] Chow NA, de Groot T, Badali H, Abastabar M, Chiller TM, Meis JF. 2019. Potential fifth clade of *Candida auris*, Iran, 2018. Emerg Infect Dis 25:1780–1781. doi:10.3201/eid2509.190686. 31310230 PMC6711235

[4] van Schalkwyk E, Mpembe R, Thomas J, Shuping L, Ismail H, Lowman W, Karstaedt A, Chibabhai V, Wadula J, Avenant T, Messina A, Govind C, Moodley K, Dawood H, Ramjathan P, Govender N, for GERMS-SA. 2019. Epidemiologic shift in candidemia driven by *Candida auris*, South Africa, 2016–2017. Emerg Infect Dis 25:1698–1707. doi:10.3201/eid2509.190040. 31441749 PMC6711229

[5] Sabino R, Veríssimo C, Pereira ÁA, Antunes F. 2020. *Candida auris*, an agent of hospital-associated outbreaks: which challenging issues do we need to have in mind? Microorganisms 8:181. doi:10.3390/microorganisms8020181. 32012865 PMC7074697

[6] Chowdhary A, Tarai B, Singh A, Sharma A. 2020. Multidrug-resistant *Candida auris* infections in critically ill coronavirus disease patients, India, April-July 2020. Emerg Infect Dis 26:2694–2696. doi:10.3201/eid2611.203504. 32852265 PMC7588547

[7] Britz E, Govender NP. 2016. Global emergence of a multi-drug resistant fungal pathogen, *Candida auris*. South Afr J Infect Dis 31:2.

[8] Kordalewska M, Perlin DS. 2019. Identification of drug resistant *Candida auris*. Front Microbiol 10:1918. doi:10.3389/fmicb.2019.01918. 31481947 PMC6710336

[9] Lockhart SR, Etienne KA, Vallabhaneni S, Farooqi J, Chowdhary A, Govender NP, Colombo AL, Calvo B, Cuomo CA, Desjardins CA, Berkow EL, Castanheira M, Magobo RE, Jabeen K, Asghar RJ, Meis JF, Jackson B, Chiller T, Litvintseva AP. 2017. Simultaneous emergence of multidrug-resistant *Candida auris* on 3 continents confirmed by whole-genome sequencing and epidemiological analyses. Clin Infect Dis 64:134–140. doi:10.1093/cid/ciw691. 27988485 PMC5215215

[10] Muñoz JF, Gade L, Chow NA, Loparev VN, Juieng P, Berkow EL, Farrer RA, Litvintseva AP, Cuomo CA. 2018. Genomic insights into multidrug-resistance, mating and virulence in *Candida auris* and related emerging species. Nat Commun 9:5346. doi:10.1038/s41467-018-07779-6. 30559369 PMC6297351

[11] Rhodes J, Abdolrasouli A, Farrer RA, Cuomo CA, Aanensen DM, Armstrong-James D, Fisher MC, Schelenz S. 2018. Genomic epidemiology of the UK outbreak of the emerging human fungal pathogen *Candida auris*. Emerg Microbes & Infect 7:43. doi:10.1038/s41426-018-0045-x. 29593275 PMC5874254

[12] Chow NA, Muñoz JF, Gade L, Berkow EL, Li X, Welsh RM, Forsberg K, Lockhart SR, Adam R, Alanio A, Alastruey-Izquierdo A, Althawadi S, Araúz AB, Ben-Ami R, Bharat A, Calvo B, Desnos-Ollivier M, Escandón P, Gardam D, Gunturu R, Heath CH, Kurzai O, Martin R, Litvintseva AP, Cuomo CA. 2020. Tracing the evolutionary history and global expansion of *Candida auris* using population genomic analyses. mBio 11:e03364-19. doi:10.1128/mBio.03364-19. 32345637 PMC7188998

[13] Govender NP, Magobo RE, Mpembe R, Mhlanga M, Matlapeng P, Corcoran C, Govind C, Lowman W, Senekal M, Thomas J. 2018. *Candida auris* in South Africa, 2012–2016. Emerg Infect Dis 24:2036–2040. doi:10.3201/eid2411.180368. 30334713 PMC6200016

[14] Govender NP, Avenant T, Brink A, Chibabhai V, Cleghorn J, Du Toit B, Govind C, Lewis E, Lowman W, Mahlangu H, Maslo C, Messina A, Mer M, Pieton K, Seetharam S, Sriruttan C, Swart K, van Schalkwyk E. 2019. Federation of infectious diseases societies of Southern Africa guideline: recommendations for the detection, management and prevention of healthcare-associated *Candida auris* colonisation and disease in South Africa. South African J Infect Dis 34. 10.4102/sajid.v34i1.163 PMC8377779 34485460

[15] Frías-De-León MG, Hernández-Castro R, Vite-Garín T, Arenas R, Bonifaz A, Castañón-Olivares L, Acosta-Altamirano G, Martínez-Herrera E. 2020. Antifungal resistance in Candida auris: molecular determinants. Antibiotics (Basel) 9:568. doi:10.3390/antibiotics9090568. 32887362 PMC7558570

[16] Maphanga TG, Naicker SD, Kwenda S, Muñoz JF, van Schalkwyk E, Wadula J, Nana T, Ismail A, Coetzee J, Govind C, Mtshali PS, Mpembe RS, Govender NP for GERMS-SA. 2021. In-vitro antifungal resistance of *Candida auris* isolates from bloodstream infections, South Africa. Antimicrob Agents Chemother 65:e00517-21. doi:10.1128/AAC.00517-21. 34228535 PMC8370198

[17] Chowdhary A, Prakash A, Sharma C, Kordalewska M, Kumar A, Sarma S, Tarai B, Singh A, Upadhyaya G, Upadhyay S, Yadav P, Singh PK, Khillan V, Sachdeva N, Perlin DS, Meis JF. 2018. A multicentre study of antifungal susceptibility patterns among 350 *Candida auris* isolates (2009–2017) in India: role of the *ERG11* and *FKS1* genes in azole and echinocandin resistance. J Antimicrob Chemother 73:891–899. doi:10.1093/jac/dkx480. 29325167

[18] Ostrowsky B, Greenko J, Adams E, Quinn M, O’Brien B, Chaturvedi V, Berkow E, Vallabhaneni S, Forsberg K, Chaturvedi S, Lutterloh E, Blog D, Bucher C, Denis RJ, Erazo R, Fernandez R, Southwick K, Zhu YC, *C. auris* Investigation Working Group. 2020. *Candida auris* isolates resistant to three classes of antifungal medications-New York, 2019. MMWR Morb Mortal Wkly Rep 69:6–9. doi:10.15585/mmwr.mm6901a2. 31917780 PMC6973342

[19] Gintjee TJ, Donnelley MA, Thompson GR, 3rd. 2020. Aspiring antifungals: review of current antifungal pipeline developments. J Fungi (Basel) 6. doi:10.3390/jof6010028. PMC7151215 32106450

[20] Pfaller M, Huband M, Flamm R, Bien P, Castanheira M. 2019. In vitro activity of APX001A (manogepix) and comparator agents against 1,706 fungal isolates collected during an international surveillance program in 2017. Antimicrob Agents Chemother 63:e00840-19. doi:10.1128/AAC.00840-19. 31182527 PMC6658749

[21] Hager CL, Larkin EL, Long L, Zohra Abidi F, Shaw KJ, Ghannoum MA. 2018. In vitro and in vivo evaluation of the antifungal activity of APX001A/APX001 against *Candida auris*. Antimicrob Agents Chemother 62. doi:10.1128/AAC.02319-17. PMC5826120 29311065

[22] Arendrup MC, Chowdhary A, Jørgensen KM, Meletiadis J. 2020. Manogepix (APX001A) in vitro activity against *Candida auris*: head-to-head comparison of EUCAST and CLSI MICs. Antimicrob Agents Chemother 64:e00656-20. doi:10.1128/AAC.00656-20. 32660998 PMC7508601

[23] Jørgensen KM, Astvad KM, Arendrup MC. 2020. In vitro activity of manogepix (APX001A) and comparators against contemporary molds: MEC comparison and preliminary experience with colorimetric MIC determination. Antimicrob Agents Chemother 64:e00656-20. doi:10.1128/AAC.00656-20. 32513793 PMC7526828

[24] Zhu Y, Kilburn S, Kapoor M, Chaturvedi S, Shaw KJ, Chaturvedi V. 2020. In vitro activity of manogepix against multidrug-resistant and panresistant Candida auris from the New York outbreak. Antimicrob Agents Chemother 64:e01124-20. doi:10.1128/AAC.01124-20. 32839219 PMC7577132

[25] Badali H, Patterson HP, Sanders CJ, Mermella B, Gibas CFC, Ibrahim AS, Shaw KJ, Wiederhold NP. 2021. Manogepix, the active moiety of the investigational agent fosmanogepix, demonstrates *in vitro* activity against members of the *Fusarium oxysporum* and *Fusarium solani* species complexes. Antimicrobial Agents Chemother 65:e02343-20. doi:10.1128/AAC.02343-20. PMC8315997 33722886

[26] Pfaller MA, Huband MD, Flamm RK, Bien PA, Castanheira M. 2021. Antimicrobial activity of manogepix, a first-in-class antifungal, and comparator agents tested against contemporary invasive fungal isolates from an international surveillance programme (2018–2019). J Glob Antimicrob Resist 26:117–127. doi:10.1016/j.jgar.2021.04.012. 34051400

[27] Shaw KJ, Ibrahim AS. 2020. Fosmanogepix: a review of the first-in-class broad spectrum agent for the treatment of invasive fungal infections. J Fungi (Basel) 6:239. doi:10.3390/jof6040239. 33105672 PMC7711534

[28] Arendrup MC, Chowdhary A, Astvad KMT, Jørgensen KM. 2018. APX001A in vitro activity against contemporary blood isolates and *Candida auris* determined by the EUCAST reference method. Antimicrob Agents Chemother 62:e01225-18. doi:10.1128/AAC.01225-18. 30104264 PMC6153824

[29] Berkow EL, Lockhart SR. 2018. Activity of novel antifungal compound APX001A against a large collection of *Candida auris*. J Antimicrob Chemother 73:3060–3062. doi:10.1093/jac/dky302. 30085167

[30] Naicker SN, Maphanga TG, Chow NA, Allam M, Kwenda S, Ismail A, Govender NP, for GERMS-SA. 2021. Clade distribution of *Candida auris* in South Africa using whole genome sequencing of clinical and environmental isolates. Emerg Microbes Infect 10:1300–1308. doi:10.1080/22221751.2021.1944323:1-24. 34176429 PMC8253216

[31] Clinical and Laboratory Standard Institute. 2012. Reference method for broth 419 dilution antifungal susceptibility testing of yeasts. 4th Informational Supplement. 420 Clinical and Laboratory Standards Institute M27-A3, Wayne, PA, USA.

[32] Pfaller MA, Diekema DJ, Jones RN, Castanheira M. 2014. Use of anidulafungin as a surrogate marker to predict susceptibility and resistance to caspofungin among 4,290 clinical isolates of *Candida* by using CLSI methods and interpretive criteria. J Clin Microbiol 52:3223–3229. doi:10.1128/JCM.00782-14. 24951808 PMC4313142

[33] Turnidge J, Kahlmeter G, Kronvall G. 2006. Statistical characterisation of bacterial wild-type MIC value distributions and the determination of epidemiological cut-off values. Clin Microbiol Infect 12:418–425. doi:10.1111/j.1469-0691.2006.01377.x. 16643517

